# A PAMPA Assay as Fast Predictive Model of Passive Human Skin Permeability of New Synthesized Corticosteroid C-21 Esters

**DOI:** 10.3390/molecules17010480

**Published:** 2012-01-05

**Authors:** Bojan D. Markovic, Sote M. Vladimirov, Olivera A. Cudina, Jadranka V. Odovic, Katarina D. Karljikovic-Rajic

**Affiliations:** 1 Department of Pharmaceutical Chemistry, Faculty of Pharmacy, University of Belgrade, Vojvode Stepe 450, 11221 Belgrade, Serbia; Email: sotevlad@pharmacy.bg.ac.rs (S.M.V.); ocudina@pharmacy.bg.ac.rs (O.A.C.); 2 Department of Analytical Chemistry, Faculty of Pharmacy, University of Belgrade, Vojvode Stepe 450, 11221 Belgrade, Serbia; Email: jodovic@pharmacy.bg.ac.rs (J.V.O.); kkrajic@pharmacy.bg.ac.rs (K.D.K.-R.)

**Keywords:** corticosteroid C-21 esters, prodrug, PAMPA, membrane retention, permeation parameter

## Abstract

The permeation properties of twenty newly synthesized α-alkoxyalkanoyl and α-aryloxyalkanoyl C-21 esters of standard corticosteroids: Fluocinolone acetonide, dexamethasone, triamcinolone acetonide and hydrocortisone were established using a PAMPA assay (70% silicone oil and 30% isopropyl myristate). The data were compared with parent corticosteroids with addition of mometasone furoate and hydrocortisone acetate. All newly synthesized corticosteroid C-21 esters have effective permeability coefficients higher then -6, mostly followed with high values of retention factors and low permeation. The examined compounds were grouped through relationship between obtained retention factors and permeation parameters (groups I–III). The classification confirmed group I (membrane retentions as well as permeation lower then 30%) for all corticosteroid standards except mometasone furoate, a potent topical corticosteroid which, with high membrane retention (81%) and low permeation (7.7%) fits into group III. The largest number of new synthesized corticosteroids C-21 esters, among them all fluocinolone acetonide C-21 esters, have high membrane retentions (32.4%–86.5%) and low permeations (1.3%–27.1%), fitting in group III. The classification was related to previously obtained anti-inflammatory activity data for the fluocinolone acetonide C-21 esters series. According to the PAMPA results the new synthesized esters could be considered as potential new prodrugs with useful benefit/risk ratio.

## 1. Introduction

Topical corticosteroids represent a significant group of drugs widely used in the therapy of skin disorders due to their anti-inflammatory and immunosuppressive actions. The applications of corticosteroids, especially to large areas, when the skin is injured, or under occlusive dressings may led to their absorption and systemic side-effects [1]. The local corticosteroids efficiency depends, at the first place, on their pharmacological potency and ability to be absorbed into the target epidermis and dermis cells [2,3,4]. Topical steroids can be divided into different groups due to their potencies. According the US classification topical corticosteroids could be classified into seven groups [5], while the British National Formulary suggests four classes [6]. The corticosteroids from the same group have similar efficiency and the similar potential to exert side effects. Thus, more potent corticosteroid product provides better therapeutic efficacy, but also more side effects. It is consider that the low-potent formulations are more suitable for application in long-term therapy, while more potent formulation are reserved for short time treatments and for use at areas such as the hands or soles, where poorly potent corticosteroids are not effective [7].

Drug skin penetration is an area of increasing interest in the pharmaceutical and cosmetic industries, but also in dermal exposure and risk assessment processes. Until today, numerous *in vivo* and *in vitro* test models have been introduced, particularly in drug discovery and development, to mimic the different biological barriers and to predict drug absorption or permeability [8,9]. Since, for *in vivo* experiments of skin penetration, either on humans or animals, ethical principles must be considered there was a need to establish *in vitro* methods/models capable for dermal absorption prediction [10,11]. The number of *in vitro* methods for evaluation drugs skin permeability employs diffusion cells which typically consist of human or animal skin clip [12,13,14]. The quantitative structure-permeability relationships (QSPRs) models as well as chromatographic methods such as immobilized artificial membrane (IAM) or biopartitioning micelar chromatography (BMC) were also applied in dermal absorption prediction [8,14,15,16], but these methodologies do not fully reflect the human skin properties.

The Parallel Artificial Membrane Permeability Assay (PAMPA) is a technique developed for the early evaluation of passive transport permeability [17]. Depending on the nature of the artificial membrane, different biological barriers (intestinal or blood-brain) can be targeted [18,19,20]. An adaptation of the PAMPA technique has been recently proposed in order to mimic a percutaneous barrier. Until today, there are only two proposed models for high-throughput screening (HTS) permeability through the skin using the PAMPA method. The first model applied silicone and isopropyl myristate (IPM), which are not natural human skin components, as an artificial membrane [21,22]. The artificial membrane composed as a mixture of isopropyl myristate (30%) and silicone (70%) allows prediction of human skin permeability, as well as the affinities of tested compounds for the *stratum corneum*, in a simple and rapid way [21,22]. The second model contains ceramide analogues, compounds similar to those present in the skin, which are responsible for the barrier function [23]. Although excised skin (human or animal) with its two-pathway mode of transport-lipophilic and hydrophilic, can be considered as the membrane of choice in permeation estimation, the difficulties in samples obtaining as well as the high variability of samples led to use of artificial membrane. PAMPA is much less labor intensive than Franz diffusion cell method using skin samples, but it appears to show similar predictability. One of the main limitations of PAMPA model is its underestimation of the compounds absorptions that are actively absorbed via drug transporters. Despite the limitation, PAMPA may serve as a very useful primary permeability screen during early drug discovery process because of its high throughput capability.

The PAMPA process is automated and commercially available and can be used as a HTS method for *in vitro* determination of drug's skin absorption potential [24,25]. In PAMPA permeability measurements different detection methods can be used: UV-Vis detection, liquid chromatography especially coupled to MS or more recently UPLC coupled to MS detection [26,27,28].

In our recent papers the studies of solvolysis of new synthesized fluocinolone acetonide C-21 esters as *in vitro* model for predicting prodrug activation [29] and their topical anti-inflammatory activity [30] were reported. The aim of this study was to established membrane retentions, permeation parameters and effective permeability coefficients using PAMPA assay for twenty newly synthesized corticosteroid C-21 esters of standard corticosteroids: fluocinolone acetonide (FA), dexamethasone (DEX), triamcinolone acetonide (TA) and hydrocortisone (H). On the basis of PAMPA data the classification of investigated corticosteroid C-21 esters in comparison with standards (FA, DEX, TA, H with addition of mometasone furoate–MF and hydrocortisone acetate–H-21-Ac) was accomplished. The classification was related to previously obtained data for anti-inflammatory activity of fluocinolone acetonide C-21 esters.

## 2. Results and Discussion

In this paper the permeability of six standard corticosteroids (FA, DEX, TA, H, MF and H-21-Ac) as well as twenty newly synthesized C-21 α-alkoxyalkanoyl and α-aryloxyalkanoyl esters of FA, DEX, TA and H were investigated with the PAMPA test. The test was performed on an artificial membrane consisting of 70% silicone oil and 30% IPM, which was originally proposed by Ottaviani and coworkers [21,22]. This PAMPA model was applied in our study to test the membrane retention and membrane permeability of investigated corticosteroids using relatively fast and easy technique. These investigations were of importance for correlation to their local anti-inflammatory activity. 

The study was performed in triplicate and the results are presented as mean values. After the PAMPA tests was performed, a HPLC method was used to measure the concentrations of the investigated corticosteroids: the initial concentration–*C*_D_(0) in the starting solution, the residual concentration–*C*_D_(t) in the donor compartment (7 h after incubation) and the transferred concentration–*C*_A_(t) in the acceptor compartment (7 h after incubation). The assay was performed using the previously described HPLC method [29] with mobile phase modification for determination of standard corticosteroids. The mobile phase modification was introduced to achieve complete separation standard corticosteroids peak from DMSO peak, which was used as cosolvent. DMSO was used as cosolvent with the aim of increasing the corticosteroid C-21 esters’ solubility. DMSO is one of the earliest and most widely studied penetration enhancers for transdermal drug delivery systems.

On the basis of the initial (100 μM) and calculated residual and transferred concentrations according to peak area comparison at zero time *t_0_*, the retention factors–*R* and permeability coefficients–log*P_e_* of investigated corticosteroids were obtained. The values of *R*, log*P_e_* as well as permeation parameters *C*_A_(*t*)/*C*_D_(0) for standards (FA, DEX, TA, H) and their twenty corresponding C-21 esters of 2-methoxypropionic (MPA), 2-ethoxypropionic (EPA), 2-phenoxypropionic (PhPA), 2-methoxybutyric (MBA) and 2-ethoxybutyric (EBA) acids obtained in PAMPA test are presented in Table 1. For the correlation, standard MF [31] as most efficient topical corticosteroid as well as H-21-Ac as drug introduced long time ago, were tested simultaneously.

**Table 1:** The values of retention factors (*R*), permeation parameters *C*_A_(*t*)/*C*_D_(0) and permeability coefficients (log*P_e_*) obtained in PAMPA test with membrane containing 70% silicone oil and 30% IPM.

		Corticosteroids	R (%)	C_A_(t)/C_D_(0) (%)	log *P_e_* (cm/s)
**Synthesized corticosteroid C-21esters**	1	FA-21-MP	34.1 ± 3.5	23.1 ± 1.4	−4.64 ± 0.17
	2	FA-21-EP	45.8 ± 4.1	22.2 ± 1.6	−4.52 ± 0.19
	3	FA-21-PhP	71.9 ± 2.8	1.3 ± 0.6	−5.68 ± 0.15
	4	FA-21-MB	47.2 ± 3.4	18.0 ± 1.3	−4.65 ± 0.12
	5	FA-21-EB	72.8 ± 2.6	7.2 ± 0.9	−4.82 ± 0.10
	6	DEX-21-MP	<1	23.8 ± 1.5	−4.88 ± 0.17
	7	DEX-21-EP	<1	31.6 ± 0.6	−4.70 ± 0.11
	8	DEX-21-PhP	86.5 ± 4.2	4.1 ± 1.1	−4.74 ± 0.13
	9	DEX-21-MB	<1	37.4 ± 1.5	−4.58 ± 0.15
	10	DEX-21-EB	11.3 ± 3.5	33.4 ± 1.2	−4.58 ± 0.13
	11	TA-21-MP	13.5 ± 4.1	38.1 ± 1.6	−4.45 ± 0.16
	12	TA-21-EP	32.4 ± 1.7	23.0 ± 1.8	−4.66 ± 0.15
	13	TA-21-PhP	51.1 ± 3.8	27.1 ± 1.4	−4.07 ± 0.14
	14	TA-21-MB	39.3 ± 4.1	16.9 ± 2.0	−4.85 ± 0.23
	15	TA-21-EB	70.1 ± 3.4	7.3 ± 1.6	−4.87 ± 0.21
	16	H-21-MP	2.6 ± 1.9	14.9 ± 1.8	−5.11 ± 0.25
	17	H-21-EP	17.2 ± 2.2	16.3 ± 1.9	−4.99 ± 0.19
	18	H-21-PhP	72.2 ± 3.1	10.6 ± 1.2	−4.58 ± 0.15
	19	H-21-MB	9.6 ± 3.2	18.5 ± 1.9	−4.97 ± 0.28
	20	H-21-EB	38.6 ± 1.8	17.6 ± 1.4	−4.77 ± 0.17
**Corticosteroid standards**	21	**FA**	<1	6.7 ± 1.2	−5.26 ± 0.15
	22	**DEX**	4.2 ± 2.0	1.3 ± 0.8	−5.99 ± 0.10
	23	**TA**	4.7 ± 2.3	10.8 ± 1.5	−5.01 ± 0.17
	24	**H**	1.5 ± 0.9	0.8 ± 0.5	−6.23 ± 0.09
	25	**H-21-Ac**	<1	10.0 ± 1.7	−5.07 ± 0.19
	26	**MF**	81.0 ± 2.9	7.7 ± 1.5	−4.10 ± 0.15

**Figure 1:**
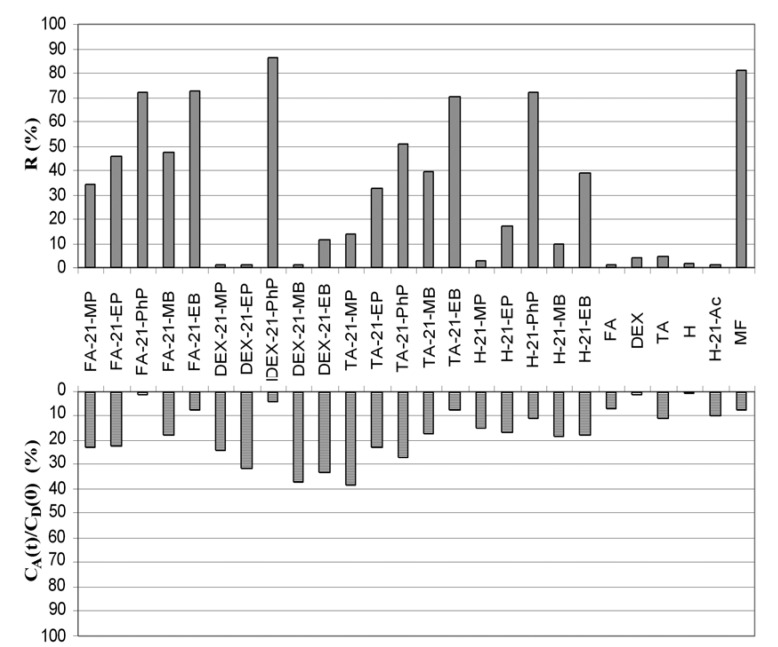
The values of retention factors (*R*) and permeation parameters *C*_A_(*t*)/*C*_D_(0) of new synthesized C-21 esters and corticosteroid standards obtained in PAMPA test.

The analogous histograms of membrane retention-retention factors (*R*) and permeation parameters *C*_A_(*t*)/*C*_D_(0) are presented at Figure 1. The largest number of new synthesized corticosteroid C-21 esters showed significantly higher membrane retention in comparison with corresponding standards confirming that their higher lipophilicity improved the parent drugs physical and pharmaceutical properties that would increase benefit/risk ratio [32]. The corticosteroid C-21 esters of PhPA and EBA (with exception of DEX-21-EB) showed comparable *R* values to MF as potent (ointment) and mid-strength (cream)–Class 2 and Class 4, respectively [5] or Class II–potent according British National Formulary [6]. The same corticosteroid could belong in different classes depending on pharmaceutical formulation, as example of MF.

Low values of permeation parameters are preferable and most of the C-21 esters of PhPA and EBA showed acceptable *C*_A_(*t*)/*C*_D_(0) values. Only two, DEX-21-EB and TA-21-PhP showed relatively higher skin permeability. In the PAMPA test results for H-21-Ac, the low membrane retention (*R* < 1%) and high permeation parameter (*C*_A_(*t*)/*C*_D_(0) = 10%), confirmed its classification as mild-Class I [6].

**Figure 2:**
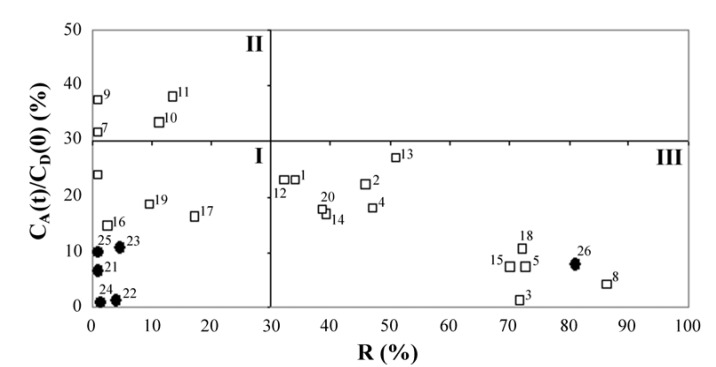
The relationship between retention factors, *R* and permeation parameters, *C*_A_(*t*)/*C*_D_(0)of examined twenty new synthesized C-21 esters and standard corticosteroids obtained in PAMPA test. ● - standard corticosteroids; □ - twenty new synthesized C-21 esters.

It can be seen (Table 1) that all new synthesized C-21 esters have log *P_e_* > −6, mostly followed with relatively high values of retention factors (*R*). The classification of examined standard corticosteroids and the twenty new synthesized C-21 esters obtained in the PAMPA test is presented at Figure 2 through the relationship between their retention factors, *R* and permeation parameters, *C*_A_(*t*)/*C*_D_(0).

Ottaviani and coworkers [21] predicted skin permeation of numerous compounds applying a new membrane (70% silicone oil and 30% IPM) in the PAMPA test and presented permeate classifications on the basis of available literature permeability coefficients through human skin, log*K_p_*. According to Ottaviani and coworkers [21], permeates can be distinguished and grouped into: Permeates with a lower permeability coefficient, *K_p_* (log*K_p_* < −6) having negligible membrane retention and low permeation (group I) and compounds with higher *K_p_* (log*K_p_* ≥ −6) having low or negligible membrane retention and high permeation (group II) or having high membrane retention and low permeation (group III).

The authors also confirmed [21] the good correlation (r^2^ = 0.81) between log*K_p_* and effective permeability coefficients (log*P_e_*) obtained after 7 h incubation time through 70% silicone–30% IPM membrane. The proposed equation for correlation between log*K_p_* and log*P_e_* for 31 investigated compounds was:

log*K_p_* = (1.34 ± 0.12) log*P_e_* + (0.28 ± 0.56) (1)

Applying this equation, in our study only the approximate log*K*_p_ values could be calculated, indicating that corticosteroid standards (FA, DEX, TA, H, H-21-Ac) with log*K_p_* < −6 (−6.77, −7.75, −6.43, −8.07, −6.51, respectively) fit in group I, while only MF had log*K_p_* > −6 (−5.21). Calculated log*K_p_* values of the new synthesized corticosteroid C-21 esters were around −6 with the exception of FA-21-PhP (log*K_p_* = −7.33). The classification (Figure 2) through the relationship between retention factors (*R*) and permeation parameters (*C*_A_(*t*)/*C*_D_(0)) confirmed group I for corticosteroid standards FA, DEX, TA, H, H-21-Ac, while MF with high membrane retention (*R* = 81%) and low permeation (*C*_A_(*t*)/*C*_D_(0) = 7.7%) fit in group III. The results for DEX obtained in our study were in good accordance to previously reported data (log*P_e_* = −5.75) for this corticosteroid by Ottaviani *et al.* [21].

For group III there is a negative relationship between membrane retention (*R*) and permeation parameter *C*_A_(*t*)/*C*_D_(0) (high membrane retention and low permeation), indicating the membrane was a trap for the investigated substances. It would be preferable that corticosteroids for local application belong to the group III where MF already belongs as a standard potent topical corticosteroid [31]. The largest number of newly synthesized corticosteroids C-21 esters (12) have high membrane retention (*R*) and low permeation [*C*_A_(*t*)/*C*_D_(0)], thus fitting in group III (Figure 2). According to this classification (group III), investigated C-21 esters could be considered as potential new prodrugs with useful benefit/risk ratios.

In our previous paper [30] the anti-inflammatory activity of new fluocinolone acetonide C-21 esters was evaluated in the test of inhibition of croton oil induced mice ear edema (4 h after application). For the same group of FA C-21 esters in our recently published paper [29] it was established that the *in vitro* proposed solvolytic model, obtained by multiple regression analysis between solvolytic rate constant and lipophilicity with anti-inflammatory activity, could be used for prediction of prodrug activation. Anti-inflammatory activity [30], represented as a percentage of reduction of mass edema, has been determined using median effective dose ED_50_ (28 μM) of FA [33]. In comparison to FA (100%) relative anti-inflammatory activities were calculated. The best result was obtained with FA-21-MP (108.12%) while comparable activities were manifested by FA-21-PhP (65%) and FA-21-EB (60%) and the lowest activity was obtained with FA-21-EP (less than 10%). The obtained activity order showed a good relationship with predicted anti-inflammatory activity, which was linked with solvolysis rate constants (K) and lipophilicity data (cLogP values ranged from 2.79 to 4.59) of FA C-21 esters [29].

Results in the PAMPA test confirmed that all investigated FA C-21 esters (compounds 1–5) have high membrane retentions (*R*) and low permeation parameters [*C*_A_(*t*)/*C*_D_(0), Table 1] and fit in group III (Figure 2). The PAMPA results indicate a possible prolonged effect of new synthesized corticosteroid C-21 esters and their *in vivo* anti-inflammatory activities should be evaluated during at least 24 h in further studies.

The major route of percutaneous transport, especially for hydrophobic molecules (such as the parent standard corticosteroids and the newly synthesized C-21 esters) is passive diffusion through the lipid matrix which can be successfully mimicked with a silicone/IPM artificial membrane in PAMPA tests. The data obtained in the PAMPA test designate that structural changes of parent standard corticosteroids (FA, DEX, TA, H) in the set of new synthesized esters would enable both, the better permeation through *stratum corneum* and higher retention in epidermis and could improve their anti-inflammatory activity.

## 3. Experimental

### 3.1. General

**Scheme 1:**
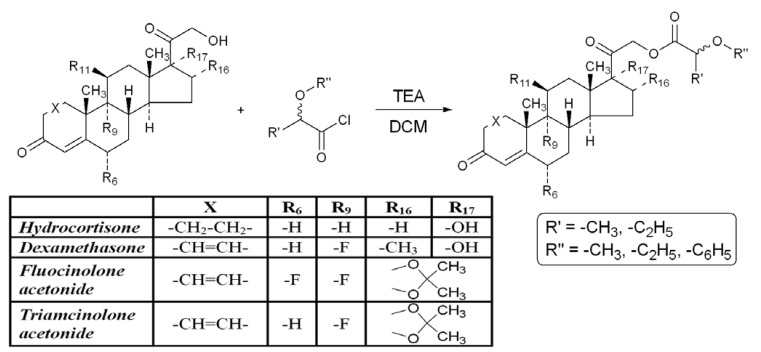
Synthesis of new C-21 α-alkoxyalkanoyl and α-aryloxyalkanoyl corticosteroid esters.

Six corticosteroid standards: fluocinolone acetonide–FA, dexamethasone–DEX, triamcinolone acetonide–TA, hydrocortisone–H, mometasone furoate–MF and hydrocortisone acetate–H-21-Ac and twenty newly synthesized corticosteroid esters: (a) FA series–fluocinolone acetonide 21-(2′-methoxy-propionate) (FA-21-MP, **1**), fluocinolone acetonide 21-(2′-ethoxypropionate) (FA-21-EP, **2**), fluocinolone acetonide 21-(2′-phenoxypropionate) (FA-21-PhP, **3**), fluocinolone acetonide 21-(2′-methoxybutyrate) (FA-21-MB, **4**) and fluocinolone acetonide 21-(2′-ethoxybutyrate) (FA-21-EB, **5**); (b) DEX series–dexamethasone 21-(2′-methoxypropionate) (DEX-21-MP, **6**), dexamethasone 21-(2′-ethoxypropionate) (DEX-21-EP, **7**), dexamethasone 21-(2′-phenoxypropionate) (DEX-21-PhP, **8**), dexamethasone 21-(2′-methoxybutyrate) (DEX-21-MB, **9**) and dexamethasone 21-(2′-ethoxybutyrate) (DEX-21-EB, **10**); (c) TA series–triamcinolone acetonide 21-(2′-methoxypropionate) (TA-21-MP, **11**), triamcinolone acetonide 21-(2′-ethoxypropionate) (TA-21-EP, **12**), triamcinolone acetonide 21-(2′-phenoxypropionate) (TA-21-PhP, **13**), triamcinolone acetonide 21-(2′-methoxybutyrate) (TA-21-MB, **14**) and triamcinolone acetonide 21-(2′-ethoxybutyrate) (TA-21-EB, **15**); (d) H series–hydrocortisone 21-(2′-methoxypropionate) (H-21-MP, **16**), hydrocortisone 21-(2′-ethoxypropionate) (H-21-EP, **17**), hydrocortisone 21-(2′-phenoxypropionate) (H-21-PhP, **18**), hydrocortisone 21-(2′-methoxybutyrate) (H-21-MB, **19**) and hydrocortisone 21-(2′-ethoxybutyrate) (H-21-EB, **20**), were examined with PAMPA test. The new corticosteroid C-21 esters were synthesized as shown in Scheme 1 according to a previously presented procedure for the synthesis of fluocinolone acetonide C-21 esters [29] and characterized in our laboratory.

### 3.2. Permeability Measurements Using the PAMPA Technique

#### 3.2.1. Chemicals

The corticosteroid standard FA was purchased from Sigma Aldrich Chemie GmbH (Steinheim, Germany), DEX, H and H-21-Ac were purchased from Galenika a.d. (Belgrade, Serbia), TA was purchased from Krka (Novo Mesto, Slovenia), MF was purchased from Schering-Plough Labo (Heist–op–den–Berg, Belgia). DMSO (>99.7%) was purchased from Acros Organics (Chemie Brunschwig AG, Basel, Switzerland). Isopropyl myristate, IPM (>95%), silicone oil (DC 200), *n*-hexane (>99.5%) were purchased from Fluka Chemie GmbH (Bruch, Switzerland) while NaH_2_PO_4_·H_2_O and Na_2_HPO_4_·7H_2_O were from Merck (Darmstadt, Germany). Methanol Chromasolv HPLC purity (Sigma-Aldrich Chemie GmbH, Steinheim, Germany) and deionised water (TKA water purification system, Niederelbert, Germany) were used throughout this study.

Phosphate buffer (pH = 7) was prepared by dissolving NaH_2_PO_4_·H_2_O (1.167 g) and Na_2_HPO_4_·7H_2_O (3.093 g) in water (1000 mL) with the fixed total concentration of 20 mM. The 5% DMSO solution were prepared dissolving DMSO in phosphate buffer. The corticosteroid solutions were prepared dissolving the investigated corticosteroid standards as well as new synthesized corticosteroid C-21 esters in 5% DMSO solution. The membrane solution (30% isopropyl myristate–70% silicone) was prepared in *n*-hexane (35% v/v). An aliquot of previously prepared mixture (3.5 mL, 7 mL silicone oil and 3 mL IPM) was transferred into a 10 mL volumetric flask, dissolved and fill up to mark with *n*-hexane.

#### 3.2.2. PAMPA Test

Permeation experiments were carried out in hydrophobic PVDF 96-well filter plates (Millipore, Molsheim, France). Each well of the donor plate was coated with 35% (v/v) liquid membrane dissolved in *n*-hexane (17 μL) for 20 min to completely evaporate the solvent. Next, in each well of acceptor plate, 5% DMSO solution (400 μL) was transferred with an automatic pipette and covered by the donor plate, creating a PAMPA sandwich. In each well of the donor plate solutions of the tested corticosteroid in 5% DMSO solution (100 μM, 300 μL) was transferred by the use of an automatic pipette. Each substance was measured in triplicate at iso-pH conditions (the same pH value in donor and receptor compartment). The donor plate was covered to prevent evaporation and the whole system set up to interact with the vibratory mixer. After 7 h, PAMPA sandwich was removed from the mixer and vibration, and using the HPLC method the concentration of the investigated substances in the donor and receptor compartments were determined [29]. The HPLC method used was as previously described [29] but with some necessary modifications. A mobile phase of methanol–water (75:25 v/v) was used for analysis of corticosteroid C-21 esters, while the mobile phase methanol–water (50:50 v/v) was used for the analysis of unesterified corticosteroids to increase their retention time and prevent overlapping with the DMSO peak. Other HPLC parameters were as previously described. The concentrations of each tested corticosteroid in the appropriate time interval in the donor and acceptor compartments were calculated using the method of comparing the peak area of the initial concentration at zero time *t*_0_.

### 3.3. Permeability Calculations

The values of permeability coefficients–log*P*_e_ and retention factors–*R* of examined corticosteroids were calculated using equations (2) and (3). The retention factor, *R* can be defined as the mole fraction retained in the membrane and in the microplates (*i.e.*, filters and plate materials):

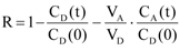
(2)
where *C*_A_(*t*)/*C*_D_(0) represents the amount of compound that reached the acceptor compartment after incubation time *t* (for *V*_A_ = *V*_D_) [21]:


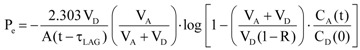
(3)

The *V*_A_ and *V*_D_ are the volumes in the acceptor and the donor wells, respectively, *A* is the filter (0.28 cm^2^), multiplied by a nominal porosity of 70% according to the manufacturer, *t* is the incubation time (s), τ_LAG_ is the steady–state time (s), *i.e.*, the time needed for the permeates concentration gradient to become stabilized, *C*_A_(*t*) is the concentration of the compound (mol·cm^−3^) in acceptor well at time *t*, and *C*_D_(0) is the concentration of the compound (mol·cm^−3^) in donor well at time 0. Steady–state time (τ_LAG_) to saturate the membranes in PAMPA are relative short compared to the total permeation time (about 20 min with unstirred plates).

## 4. Conclusions

The topical corticosteroids’ efficiency in the first place depends on their pharmacological potencies and their absorption/retention into the target epidermis. The permeation of twenty newly synthesized corticosteroid C-21 esters in comparison with six standard corticosteroids was tested with a PAMPA test using a membrane containing 70% silicone oil and 30% IPM. The majority of the newly synthesized corticosteroid C-21 esters showed higher membrane retentions and lower permeation parameters than the corresponding standard corticosteroids and could be classified into group III of permeates.The best membrane retention with lowest permeation was displayed by the new synthesized corticosteroid C-21 esters of EBA and PhPA. These esters have similar permeation properties to MF, a potent corticosteroid standard. On the basis of PAMPA results it could be assumed that the newly synthesized corticosteroid C-21 esters have favourable properties exerting local anti-inflammatory activity in comparison with standards that would be accompanied with minimal systemic side effects and useful benefit/risk ratios. The data obtained in the PAMPA test as a high-throughput screening method indicate possible prolonged effects of the newly synthesized corticosteroid C-21 ester prodrugs.
